# A Study on the Compatibility of a Food-Recording Application with Questionnaire-Based Methods in Healthy Japanese Individuals

**DOI:** 10.3390/nu16111742

**Published:** 2024-06-02

**Authors:** Katsumi Iizuka, Kanako Deguchi, Chihiro Ushiroda, Kotone Yanagi, Yusuke Seino, Atsushi Suzuki, Daisuke Yabe, Hitomi Sasaki, Satoshi Sasaki, Eiichi Saitoh, Hiroyuki Naruse

**Affiliations:** 1 Department of Clinical Nutrition, Fujita Health University, Toyoake 470-1192, Japan; kanasakuran@gmail.com (K.D.); chihiro.ushiroda@fujita-hu.ac.jp (C.U.); 2 Health Management Center, Fujita Health University, Toyoake 470-1192, Japan; yanagi-k@fujita-hu.ac.jp (K.Y.); hnaruse@fujita-hu.ac.jp (H.N.); 3 Department of Endocrinology, Diabetes, and Metabolism, Fujita Health University, Toyoake 470-1192, Japan; seinoy@fujita-hu.ac.jp (Y.S.); aslapin@fujita-hu.ac.jp (A.S.); 4 Department of Diabetes, Endocrinology and Metabolism and Department of Rheumatology and Clinical Nutrition, Gifu University Graduate School of Medicine, Gifu 501-1194, Japan; ydaisuke@kuhp.kyoto-u.ac.jp; 5 Center for One Medicine Innovative Translational Research, Gifu University, Gifu 501-1194, Japan; 6 International Medical Center, Fujita Health University Hospital, Toyoake 470-1192, Japan; sasakih@fujita-hu.ac.jp; 7 Department of Social and Preventive Epidemiology, School of Public Health, The University of Tokyo, Tokyo 113-0033, Japan; stssasak@m.u-tokyo.ac.jp; 8 Department of Rehabilitation Medicine I, Fujita Health University, Toyoake 470-1192, Japan; esaitoh1@me.com; 9 Department of Medical Laboratory Science, Fujita Health University Graduate School of Health Sciences, Toyoake 470-1192, Japan

**Keywords:** food frequency questionnaire, food frequency questionnaire based on food groups, brief self-administered diet history questionnaire, food-recording application

## Abstract

In Japan, nutritional guidance based on food-recording apps and food frequency questionnaires (FFQs) is becoming popular. However, it is not always recognized that different dietary assessment methods have different nutritional values. Here, we compared the compatibility of dietary intake data obtained from an app with those obtained from FFQs in 59 healthy individuals who recorded information regarding their diet for at least 7 days per month using an app developed by Asken (Tokyo, Japan). The diurnal coefficient of variation in total energy and protein intake was 20%, but those for vitamins B_12_ and D were >80%, reflecting the importance of 7 days of recording rather than a single day of recording for dietary intake analyses. Then, we compared the results of two FFQs—one based on food groups and one based on a brief self-administered diet history questionnaire—for 7 days, as recorded by the app. There was a correlation coefficient of >0.4 for all the items except salt. Regarding the compatibility between the app and FFQs, the percentage errors for total energy and nutrients were >40–50%, suggesting no agreement between the app and the two FFQs. In conclusion, careful attention should be paid to the impact of different dietary assessment methods on nutrient assessment.

## 1. Introduction

The methods of performing dietary intake surveys on an outpatient basis include the use of dietary records, 24 h dietary recalls, the photographic method, food frequency questionnaires (FFQs), and diet histories [1,2,3]. Because the nature of the diet is dependent on culture and lifestyle, each country has developed its own versions for tracking dietary intake [4].

The FFQ is a semiquantitative assessment of food intake [1,5,6,7,8], and FFQs are widely used to analyze the relationships between food consumption and disease in population studies [1]. The challenge of documenting and comparing diverse dietary habits within and across cultures necessitates the development and use of valid and reproducible FFQs in culture-specific population research to ensure the accurate estimation of dietary intake [2]. A key advantage of FFQs is their low cost [1]. In Japan, the brief self-administered diet history questionnaire (BDHQ) and the FFQ based on food groups (FFQg) are two commonly used questionnaires [6,7,8]. The BDHQ consists of approximately 80 questions regarding the participant’s dietary habits during the preceding month and is used to calculate the intake of 58 foods and more than 100 nutrients [6,7]. The FFQg is an assessment of food intake that comprises 20 questions regarding the participant’s diet during the preceding month [8].

Nutrition applications (apps) are excellent tools for tracking progress because they permit the storage of continuous food records on a single device [8,9,10]. The performance of apps varies according to their photo analysis function, barcode analysis function, and the number of foods referenced [9,10,11]. It has previously been reported that, compared to an FFQ, the GB HealthWatch (San Diego, CA, USA) mobile app underestimates the 3-day average intake of most macro- and micronutrients (by 5% for total calories, 19% for cobalamin, and 33% for vitamin E) [12], and the mean correlations between the values generated using these two methods were found to be 0.87 for macronutrients and 0.84 for micronutrients [12]. However, despite their widespread use, apps have rarely been compared with existing measurement methods.

It was argued that apps can be used to store daily food records, including photographs, and are therefore suitable for studying diurnal variations in nutrients [13]. We expected that some people would prefer to use an app rather than complete a relatively time-consuming questionnaire. In addition, because apps are widely used worldwide, we considered that a comparison of the results obtained using two different FFQs (FFQg and BDHQ) and an app (Asken) mostly used in Japan would assist dieticians in providing nutritional guidance for patients. In general, apps estimate total energy and nutrients over a single day, and the amount of nutrient intake with greater diurnal variation is not adequately estimated.

With the ongoing digitization of medical data around the world, there are already plans to attempt dietary assessment not only through food-recording apps, but also through web-based FFQs. In Japan, too, nutritional guidance in many clinics is based not only on traditional dietary assessment using food diary records, but also on nutritional assessment using food-recording applications and food intake frequency surveys. However, because dietitians are not fully aware that the calculation results will change if dietary intake assessment methods are changed, nutritional guidance is currently provided in accordance with guideline targets even when calculated using different methods. Therefore, we considered that it is necessary to compare the characteristics of dietary assessment methods used in Japan.

In this study, we considered the superiority of 7 days of recording to a single day of recording. To estimate the diurnal variation in each nutrient, we first analyzed the diurnal variation in nutrients using an app (Asken), to assess its suitability for this purpose. Next, we compared the results obtained using the app to those obtained using two different FFQs (FFQg and BDHQ). By clarifying the compatibility between the results obtained using two different FFQs (FFQg and BDHQ) to those obtained using the app, the present study should help determine which would be most suitable for use in future research studies and for nutritional guidance.

## 2. Materials and Methods

### 2.1. Participants

**Figure 1:**
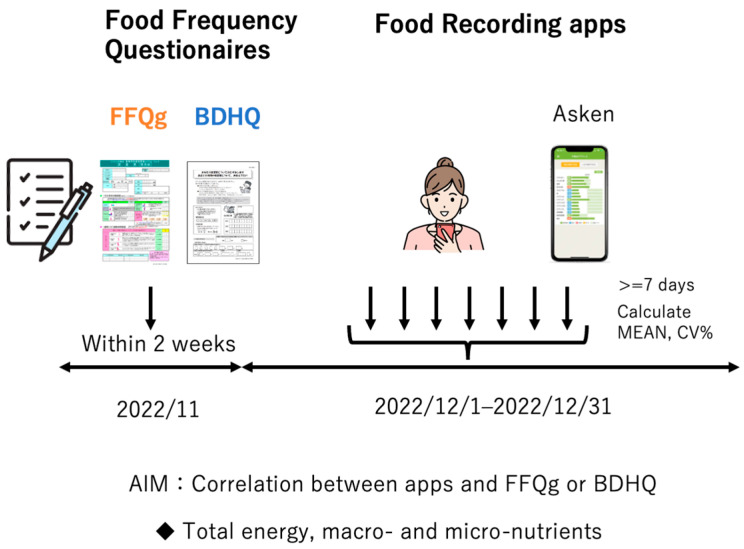
Study design. FFQ, food frequency questionnaire; FFQg, FFQ based on food groups; BDHQ, brief self-administered diet history questionnaire.

Individuals who met the following criteria were recruited: underwent a medical examination, were between the ages of 20 and 60 years, had not been diagnosed with any medical conditions, and had an average body mass. Ninety-eight average-weight men and women who underwent physical examinations and had not been diagnosed with any medical condition were invited to participate in the study. Of these, 73 underwent FFQg- and BDHQ-based investigations in November 2022. At the study site, the participants were given an explanation of the nutrition app (Asken Inc., Tokyo, Japan) and then were asked to use that app to record the details of their meals between 1 December and 31 December 2022. For those who kept records for >7 days, the 7-day mean values obtained using the app, FFQg, and BDHQ were compared (Figure 1).

**Figure 2:**
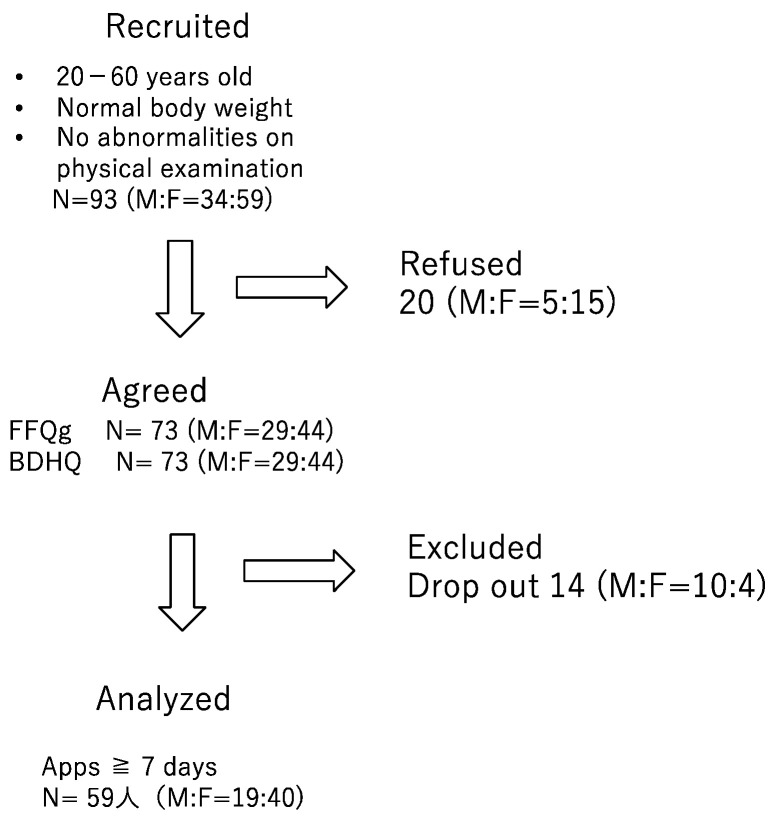
Inclusion criteria.

Seventy-three participants consented to participate in this study comparing the data obtained from the FFQs to those obtained with the app (Asken), and the results obtained using the FFQg, BDHQ, and app (Asken) were compared for 59 individuals who recorded data using the app (Asken) for at least 7 days (Figure 2). The study was approved by the Ethics Committee of Fujita Medical College (approval number: HM23-111, approved on 3 July 2023).

Of the 93 participants who were recruited, 73 consented to participate in the study, but only 59 were able to properly record the data for more than 7 days, which was needed for our analysis.

Table 1 shows a comparison of the app (Asken), FFQg, and BDHQ.

### 2.2. FFQg and BDHQ

The FFQg and BDHQ were completed between 15 November and 30 November 2022. The FFQg is based on 29 food groups and 10 styles of cooking and was validated by comparing the 7-day food records for individuals aged 19–60 years. Questions were asked regarding staple foods and main dishes, as well as cooking methods, such as rice dishes, fried dishes, simmered dishes, stir-fried dishes, and soups. The correlations between the results of the FFQg and 7-day records were previously found to average 0.4–0.5 for energy and macronutrient intake [8]. The FFQg takes 15–20 min to complete.

The BDHQ is a self-administered diet history questionnaire developed by Sasaki et al. [6,7]. The BDHQ requires 20 min to complete and consists of the following five sections: (i) intake frequency of 46 food and nonalcoholic beverage items; (ii) daily intake of rice, including the type of rice (refined or unrefined) and miso soup; (iii) frequency of alcoholic beverage consumption and amount consumed for five types of alcoholic beverages; (iv) usual cooking methods; and (v) general dietary behavior. On the basis of the answers to the questions regarding the meals that the participants ate during the preceding month, the results summarize the intake of approximately 100 nutrients and 58 food items. No statistically significant differences were noted between a 16-day dietary record and the BDHQ with respect to half of the food items [6,7].

### 2.3. Food-Recording Application (Asken)

**Figure 3:**
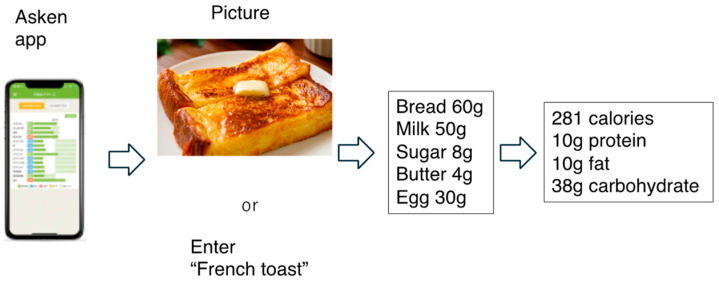
App estimate of total energy and nutrients based on recipes.

We have previously reported the accuracy of two apps (Asken and Calomeal) for recording various types of meals [9]. These apps can also recognize foods based on photographs and barcodes. Asken allows not only the manual input of meal contents but also input by means of photographs and barcodes. The macronutrient and micronutrient contents can also be calculated. For example, if you enter French toast in the app (Asken), the nutritional content is analyzed from the recipe for one serving and calculated to be 281 calories, 10 g of protein, 10 g of fat, and 38.2 g of carbohydrates (Figure 3). A correlation coefficient of 0.9 was calculated for the relationship between the app data and those collected using the 1-day dietary record method, with respect to energy intake and the intakes of the three macronutrients [14]. Thus, this method is similar to the use of paper records, but a major difference is that the nutritional value of the food is based on the food recipe rather than being determined by a nutritionist.

The app estimates total energy and nutrients based on recipes by registering foods by name or photo.

In the present study, we did not set any restrictions on these functions and allowed the participants to use their preferred method. We asked those who completed the FFQg and BDHQ surveys to record their food data in the app between 1 December and 31 December. While meal-recording applications are usually evaluated on a single-day basis, we took an average of at least seven days of records and made an evaluation. The data for participants who recorded their meals for >7 days were analyzed.

### 2.4. Statistical Analysis

The data are presented as the means ± standard deviations (SDs). We compared the app data with the FFQg and BDHQ data using a one-way analysis of variance and multiple comparisons for paired samples. We evaluated the relationships between the app results and those obtained using the FFQg or the BDHQ by calculating Pearson’s correlation coefficients. Then, we calculated the bias, agreement of the limit, and percentage error data and performed a Bland-Altman analysis. The percentage error was calculated as 2 × SD divided by the mean of the FFQg or BDHQ results. The upper and lower limits of agreement were calculated as the mean difference ± 1.96 SD. The proportional error between the two methods was evaluated using regression analysis. All the statistical analyses were conducted using Statistical Package for Social Science software (version 28.0.0.0) (IBM Inc., Armonk, NY, USA). We considered *p* < 0.05 to indicate statistical significance.

## 3. Results

### 3.1. Background of the Participants

As mentioned in the Materials and Methods section, 59 participants (19 men and 40 women) completed the two questionnaires correctly and used the app for ≥7 days. Perhaps because the study targeted individuals with no abnormalities detected on their medical examinations, 70% of the respondents were aged ≤40 years, and the mean age of the participants was 35.6 ± 9.6 years. Their BMI was 20.7 ± 1.5 kg/m^2^, which is considered normal (Table 2). The duration of use of the app was 16.8 ± 9.5 days (Table 2).

### 3.2. The Diurnal Variations in Lipid and Storable Vitamin Consumption Were the Largest

**Figure 4:**
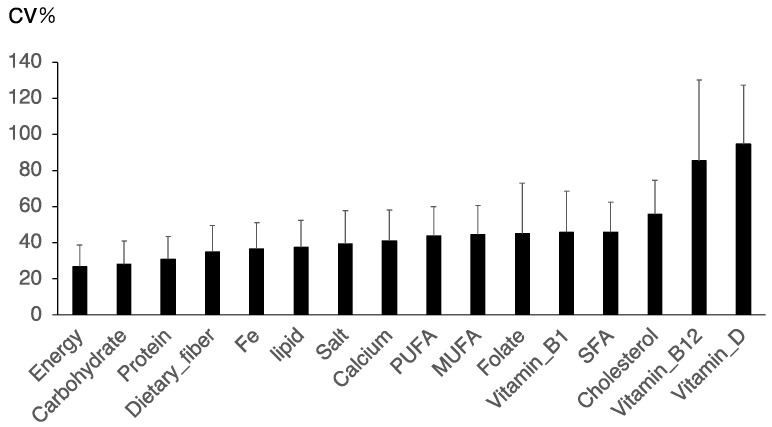
Diurnal variations in total energy and nutrient intake. The coefficient of variation (%) for the intake of each nutrient, obtained from the dietary records, was calculated using the food-recording app (Asken).

There is typically daily variation in the foods consumed by individuals, which may be reflected in the daily variation in nutrient intake. Although the FFQg can be used to determine the frequency of intake of certain foods, it is challenging to characterize such variations using this tool. Therefore, we calculated the coefficients of variation (%CVs) for the intake of nutrients using dietary records (Figure 3). We obtained app data that were recorded over 7 days and compared the daily variation in total energy intake and the intakes of the three macronutrients, trace elements, and some vitamins. The daily variations in total energy, carbohydrate, and protein intake were approximately 20–30%; however, the variations in the intake of lipids, including saturated fatty acids (SFAs), monounsaturated fatty acids (MUFAs), polyunsaturated fatty acids (PUFAs), and vitamins, were greater (40%), and those in vitamin B_12_ and vitamin D were >80% (Figure 4). Thus, there was significant variation in the daily intake of storable nutrients such as lipids and vitamins.

### 3.3. Comparison of the Energy and Nutrient Intakes Measured Using the App, the FFQg, and the BDHQ

Next, we compared the participants’ energy and nutrient intakes (total energy, the three macronutrients, trace elements, and some vitamins) obtained using the app to those obtained using the FFQg and BDHQ using one-way ANOVA with Tukey post hoc comparisons (Table 3). A comparison of the mean values obtained using the app (Asken), FFQ, and BDHQ revealed that the energy, carbohydrate, protein, fat, calcium, and iron intakes calculated using each method were, at most, 10% higher or lower, which do not represent clinically significant differences. However, for folate, SFAs, MUFAs, and PUFAs, differences of approximately 20% were obtained, and the dietary fiber intake reported using the app was much greater than that reported using the FFQg or BDHQ (*p* < 0.0001 vs. the app) (Table 3). The intakes of vitamin B_12_ and D reported using the BDHQ were significantly greater than those reported using the FFQg or the app (*p* < 0.0001 vs. the app). In contrast, the intakes of vitamin B12 and D reported using the FFQg were similar to those reported using the app (Table 3). The cholesterol intake reported using the app was lower than that reported using the FFQg, which was in turn lower than that reported using the BDHQ.

### 3.4. Relationships among the Energy and Major Nutrient Intakes Estimated Using the App (Asken), FFQg, and BDHQ

Next, we calculated the correlations among the energy and nutrient intakes estimated using the app (Asken), FFQg, and BDHQ and analyzed the factors influenced by those (Table 4).

The correlation coefficients for the FFQg and app (Asken) data were greater than 0.4 for most of the nutrients; however, the between-method correlation coefficients for MUFAs, PUFAs, vitamin D, and salt were weaker, at <0.4 (Table 4).

Regarding the relationships between the intakes assessed using the BDHQ and the app (Asken), almost all nutrients had correlation coefficients greater than 0.4; however, cholesterol and salt had weaker correlations (0.3–0.4) (Table 4). Thus, there were good correlations between the data obtained using the FFQg and the app (Asken) and between the BDHQ and the app (Asken) for most of the nutrients, except salt.

### 3.5. Verification of the Compatibility of the App (Asken) with the FFQg or BDHQ Using Bland-Altman Analysis

**Figure 5:**
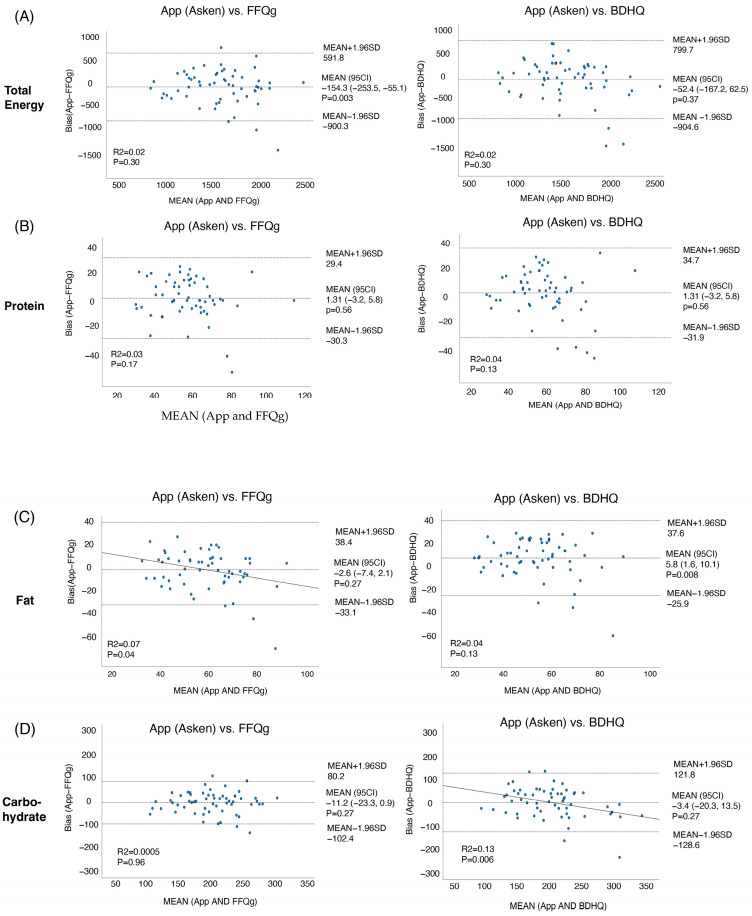

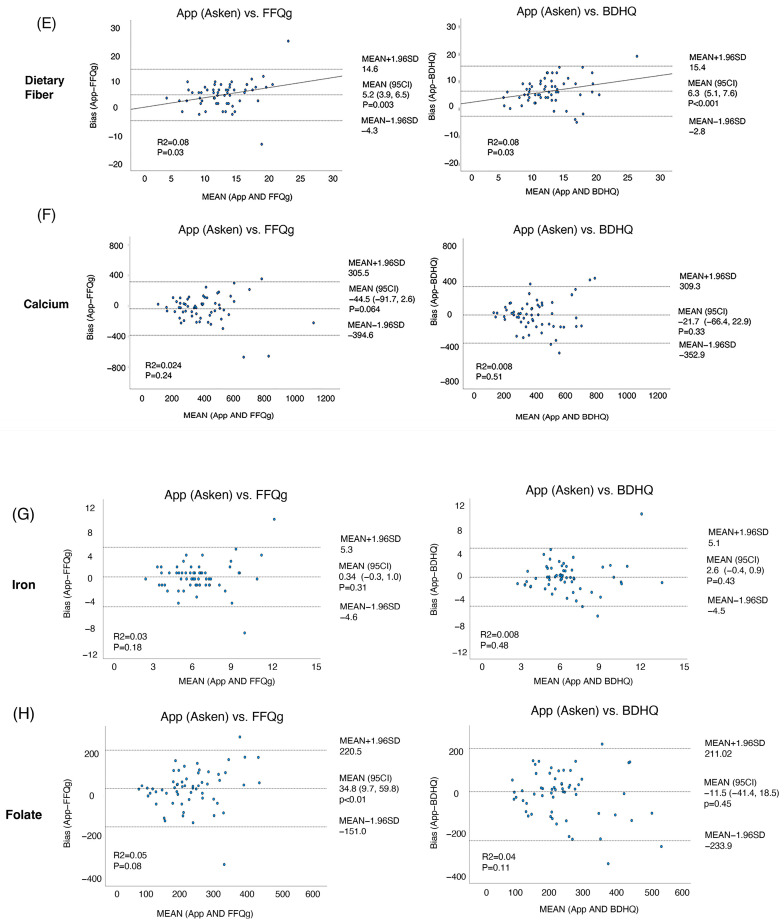
Bland–Altman plots for comparisons between the app and FFQg and between the app and BDHQ. Bland–Altman analysis was performed using SPSS software. The *X*-axis represents the mean of the app (Asken) and that of the FFQg or BDHQ. The *Y*-axis represents the difference between the app (Asken) and the FFQg or BDHQ. (**A**) total energy, (**B**) protein, (**C**) Fat, (**D**) carbohydrate, (**E**) dietary fiber, (**F**) calcium, (**G**) Iron, (**H**) Folate.

There are no ideal means of accurately assessing food intake; therefore, the compatibility of the app with the currently used FFQg and BDHQ was evaluated using the Bland–Altman method (Figure 5A–H). First, the comparison of the FFQg with the app revealed significant fixed biases of −154.3 (95% CI: −253.5, −55.1) and 5.2 (95% CI: 3.9, 6.5) for total energy and dietary fiber, respectively. However, no significant fixed bias was identified for the other nutrients (protein, fat, carbohydrate, calcium, iron, and folate). Proportional bias was identified with respect to fat and dietary fiber. In contrast, the comparison of the data obtained using the BDHQ and the app revealed fixed biases of 5.6 (1.6, 10.1) and 6.3 (5.1, 7.6) for fat and dietary fiber, respectively (Figure 5). There was a proportional bias for carbohydrates and dietary fiber. Thus, comparisons of the app vs. the FFQg or BDHQ showed systematic and proportional errors with respect to some nutrients, owing to differences in the measurement methods.

**Figure 6:**
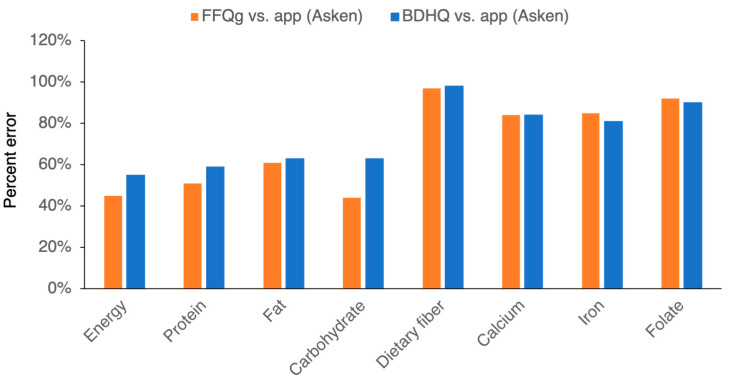
There was a lack of agreement between the app and the FFQg and between the app and the BDHQ. The percentage error was calculated as 2× precision (SD of difference between app (Asken) and FFQg or app (Asken) and BDHQ) divided by the bias (mean of the difference between app (Asken) and FFQg or app (Asken) and BDHQ).

Finally, we calculated the percentage errors to evaluate the compatibility of the methods assessed. The percentage errors for total energy, protein, fat, and carbohydrate wereapproximately 40–60% for the comparisons of the app and FFQg data (Figure 6), and the percentage errors for total energy, protein, fat, and carbohydrate were approximately 50–60% for the comparisons of the app and FFQg data. The dietary fiber, calcium, iron, and folate percentage errors were much greater (>60%) for the comparisons with both the FFQg and BDHQ. Thus, the measurement errors for these nutrients were very high (>40%), which implies that the compatibility of the app with the FFQs is low.

## 4. Discussion

In this study, total energy and nutrients were analyzed based on seven days of food records measured by an app (Asken) and compared with the results of two questionnaires. Although meal-recording applications are usually used to evaluate single-day records, it is expected that there will be diurnal variation, depending on the nutrients (fat-soluble vitamins and trace elements) ingested. First, to clarify the significance of analyzing dietary records recorded over a 7-day period rather than over a single day, we examined the diurnal variation in nutrient intake. Compared to those of energy, protein, and carbohydrate intake, the diurnal variations in vitamin B_12_ and vitamin D were much greater. These findings suggested that fat-soluble vitamins and trace elements, which can be stored, should be evaluated based on the total amount consumed over a 7-day period, not a daily record. The results measured by the app correlated with those analyzed by the BDHQ and the FFQ, but the correlations for most nutrients except salt were greater than 0.4. The amount of nutrients measured by the app was correlated with the amount of nutrients measured by the FFQg and the BDHQ. Finally, the Bland–Altman analysis revealed that the percentage error of energy and macronutrients between the app and FFQg or the app and BDHQ was 40–60%. These findings suggested that there was no clinical agreement between the app and the FFQg or BDHQ. Therefore, the app and the FFQs (FFQg and BDHQ) are different tests and should be used in a manner that exploits their respective characteristics.

It is not possible to estimate nutrient excesses or deficiencies simply by examining dietary records for a single day. The reason for this is that some nutrients are consumed daily, while others are consumed only once or twice a week. Some reported that the coefficients of within-individual variation in vitamin D, vitamin A, n-3 PUFA, and vitamin B_12_ were much greater than those of protein and carbohydrate [15]. Consistent with this, our study also supported that the intakes of storable nutrients such as vitamin D and vitamin B_12_ showed the most significant variation in daily dietary frequency. Water-soluble vitamins are usually excreted in the urine in excess, but fat-soluble vitamins accumulate in the liver and fatty tissues when taken in excess [16,17]. Vitamin B_12_ is an exception to this rule. Absorbed vitamin B_12_ is bound to transcobalamin II, which largely protects it from excretion. It is stored in a range of tissues in the body, approximately 50% of which are in the liver. Storage of vitamin B_12_ can usually maintain physiologic requirements for 3 to 5 years, even if vitamin B_12_ intake ceases [16]. The blood half-life of 25(OH) D (vitamin D) is approximately three weeks to 30 days, but it can also be stored in adipose tissue [17]. Since nutrients that accumulate in the body do not necessarily need to be consumed daily, it makes sense that there is a large diurnal variation. Therefore, given the diurnal variation in vitamin B_12_ and D, it is clear that a 7-day food record should be taken, not just a 1-day record.

The amount of fiber intake as assessed by the app (Asken) was greater than that assessed by the BDHQ or FFQg. Regarding dietary fiber, the correlation between the app and the FFQg or BDHQ was moderate, but the amount of dietary fiber estimated by the app (Asken) tended to be greater than that estimated by the FFQg and BDHQ. Moreover, the Bland-Altman plot also showed that the app (Asken) overestimated dietary fiber intake, due to fixed and proportional bias, compared with the FFQg or the BDHQ. In past reports, the average values for paper records were approximately the same as those for the BDHQ or FFQg [5,7]. Paper-based records may appear to be the same method as the app (Asken), but this is a major difference. In terms of nutritional assessment, paper records are evaluated by a skilled nutritionist, while the app calculates nutritional values based on food recipes. The differences between the app and other evaluation methods should be acknowledged.

Simple correlation analyses revealed that good correlations were generally observed for the intakes of total energy and many nutrients, except for salt and cholesterol.

Regarding salt intake, a systematic review reported poor agreement between estimates from the FFQg and those based on 24 h urine collection [18]. We have already reported that the app (Asken) cannot accurately measure salt content in experiments with test meals that already have known salt content [9]. Therefore, it is considered that the use of urinary Na is better than a medical interview regarding dietary salt.

Regarding cholesterol, the estimates of cholesterol intake provided by the app (Asken) were lower than those provided by the FFQs. Compared to paper records, the FFQg tended to provide higher estimates of cholesterol (310 ± 142 vs. 294 ± 112 mg). The estimate of vitamin B_1_ was low in the BDHQ, while the estimates of vitamins B_12_ and D were high. This trend was also observed for paper records and the FFQg. Therefore, it should be recognized that some measurement methods are subject to inherent errors.

Regarding the number of days on which measurements were taken, we set the number of days as seven days or longer, but 17% of all respondents recorded up to 7 days, 9% up to 8 days, 8% up to 9 days, and 6% up to 10 days, or approximately 40% of all respondents recorded up to 10 days. With 60% of the respondents using the app for ten days or more, more than half could use the food-recording app as a habit. Adding more than seven days was considered to have little effect on the results. The psychological burden is different if the subjects must measure every day without failing or if the subjects only had to record their dietary intake for one week out of 30 days. In some previous studies, three days of data were accumulated and compared to those of the FFQg and paper records [19]. Given the daily fluctuations in nutrient intake in an individual’s diet, it would be helpful if the meal-recording application also displayed a summary of 7 days’ worth of data, especially for fat and vitamins, for which individuals are often deficient.

In this study, total energy and the three major nutrients in the FFQg and app (Asken) or the BDHQ and app (Asken) were determined to be incompatible at 40–60%. These results indicate that the app (Asken) is not compatible with the BDHQ or FFQg. For agreement between the FFQg and BDHQ, compatibility is usually considered within a 20% error [12]. These differences may be because of differences in the methods used for measurement. Since the FFQg and BDHQ are evaluated based on personal memory and the app (Asken) calculates nutrient content from recipes based on dietary records, it is perhaps not surprising that there are differences. Moreover, the FFQg and 24 h dietary recalls underestimate the amount of energy and protein consumed, compared with the doubly labeled water method [20,21,22], which suggests that the FFQg, BDHQ, and app (Asken) are not tests that can determine absolute dietary intake. Since the doubly labeled water method is accurate but cannot be used in daily clinical practice, it is realistic to perform longitudinal assessments with a thorough understanding of the advantages and disadvantages of methods that can be used in daily practice (FFQs, 24-h dietary recalls, apps, paper records). Notably, this study was not intended to determine the superiority of FFQs or the app (Asken).

Possible reasons for the lack of compatibility between FFQs and Apps are as follows: FFQs and dietary history methods are considered suitable for the evaluation of habitual eating. On the other hand, as the present study revealed, the accumulation of records from a food-recording application allowed the diurnal variation in diet to be evaluated even with a 7-day record. If long-term records can be maintained over a period of several months, the meal history application could potentially be used to evaluate habitual eating in addition to the direct evaluation of what was eaten, which is one of the advantages of the meal history method.

Finally, as described above, all methods tend to underestimate the amount of food intake, including total energy. This finding is consistent with a previously reported paper [23]. In the comparison between the 7-day recording method and the doubly labeled water method, the reported energy intake (6.98 ± 1.58 MJ/d) was significantly lower than the energy expenditure (9.00 ± 2.08 MJ/d) and represented 79.8 ± 17.6% of the total expenditure [23]. Others reported that underreporting was 37 ± 16%. These findings suggested that precisely validating the amount of daily food intake is difficult and that only comparisons within that index (e.g., individual before/after comparisons, cross-sectional studies of populations) should be evaluated, along with changes in body weight and biomarkers.

A limitation of the study is that it first included individuals whose weight was within the normal range and did not include obese or underweight individuals. Therefore, whether these results apply to persons with nutritional disorders was not examined in this study and should be examined in the future. When using the questionnaire method, individuals with obesity may underreport, which may lead to discrepancies with the results of the app method. Furthermore, it should be recognized that the best affinity for the app method is among women and younger age groups. The participants were primarily women with a 1:2 male-to-female ratio. In our previous paper, we also conducted a study of a nutrition app, and most of the participants were women [9]. Although this is a pilot study with a small number of participants, it would be important to analyze the effects of sex on the results of future studies with a larger number of participants and by sex [24].

## 5. Conclusions

In conclusion, compared to FFQs, the app is based on records and can therefore measure diurnal variation relatively accurately. There was diurnal variation in dietary intake, especially for vitamins D and B_12_, consistent with the length of the storage period. The BDHQ and FFQg analyses correlated well with the app results for total energy and macronutrients, but salt intake showed no correlation or a low correlation. The Bland–Altman analysis revealed no agreement between the app, FFQg, and BDHQ. These results suggest that the app has the advantage of capturing diurnal variation but is incompatible with the results obtained with the FFQs. With the digitalization of healthcare, the use of food-recording apps and web-based FFQs in nutritional guidance is expected to become even more important. It is important to recognize that different methods of assessing diets may result in different assessments of nutrient and energy content. The results obtained from the FFQg and food-recording apps should not be confused.

## Figures and Tables

**Table 1:** Comparison of the app (Asken), FFQg, and BDHQ.

	Apps (Asken)	FFQg	BDHQ
Character	Each meal record was entered into the app or photographed and analyzed by the app (duration was **between a week and a month, 40% between 7–10 days**).	Calculated according to how many times and how much of what food was eaten in **a month**	Calculated according to how many times and how much of what food was eaten in **a month**
Duration	From a week to a month	One time only	One time only
Time required for one time	2–3 min per one time	15 min	20 min
Depend on Memeory	NO	YES	YES

App (Asken), food-recording application manufactured by Asken Inc. (Tokyo, Japan); FFQg, food frequency questionnaire based on food groups; BDHQ, brief self-administered diet history questionnaire.

**Table 2:** Characteristics of the participants.

	Total (n = 59)
M:F	20:39
Age (y.o.)	35.6 ± 9.6
Height (cm)	163.7 ± 7.1
BW (kg)	55.7 ± 6.9
BMI	20.7 ± 1.5
Numbers of Examinations (day)	16.8 ± 9.5

**Table 3:** Total energy and nutrient intakes estimated using the app (Asken), FFQg, and BDHQ.

	App (Asken)	FFQg	BDHQ	*p* Value
Energy (kcal)	1526.9 ± 381.3	1681.2 ± 427.6 **	1579.4 ± 493.9	** *p* < 0.01 vs. App
Protein (g)	59.2 ± 15.8	59.5 ± 18.3	57.8 ± 18.9	N.S.
Fat (g)	57.1 ± 14.5	59.7 ± 18.7	51.2 ± 17.3 **	** *p* < 0.01 vs. App
Carbohydrate (g)	198.6 ± 50.8	209.7 ± 50.5	202.0 ± 70.4	NS
Dietary fiber (g)	15.5 ± 5.1	10.3 ± 3.9 ****	9.2 ± 4.0 ****	**** *p* < 0.0001 vs. App
Calcium (mg)	383.6 ± 180.4	427.2 ± 205.4	404.4 ± 167.4	N.S.
Iron (mg)	6.4 ± 2.6	6.1 ± 2.2	6.2 ± 2.4	N.S.
Salt (g)	8.02 ± 2.02	7.6 ± 2.8	8.6 ± 2.6	N.S.
Vitamin B_1_ (mg)	0.93 ± 0.42	0.92 ± 0.30	0.66 ± 0.24 ****	**** *p* < 0.0001 vs. App
Folate (μg)	242.2 ± 106.3	207.4 ± 86.8	253.6 ± 127.2 *	* *p* < 0.05 vs. App
Vitamin B_12_ (μg)	4.40 ± 2.49	4.03 ± 2.04	6.15 ± 3.12 ***	*** *p* < 0.001 vs. App
Vitamin D (μg)	4.89 ± 2.71	4.01 ± 2.27	8.46 ± 4.94 ****	**** *p* < 0.0001 vs. App
Cholesterol (mg)	267.6 ± 89.0	294.6 ± 96.5 ***	339.6 ± 130.7 ***	*** *p* < 0.001 vs. App
SFAs (g)	16.5 ± 5.1	19.4 ± 6.4 **	14.1 ± 5.1 **	** *p* < 0.01 vs. App
MUFAs (g)	21.0 ± 5.9	21.1 ± 6.4	18.8 ± 6.7 *	* *p* < 0.05 vs. App
PUFAs (g)	10.3 ± 3.1	12.1 ± 4.3 **	12.0 ± 4.3 **	** *p* < 0.01 vs. App

Abbreviations: SFAs, saturated fatty acids; MUFAs, monounsaturated fatty acids; PUFAs, polyunsaturated fatty acids.

**Table 4:** Results of the Pearson correlation analysis of the results obtained using the app (Asken) and the FFQg or BDHQ.

	FFQg vs. App (Asken)				BDHQ vs. App (Asken)			
		95% CI				95% CI		
	R	Lower	Upper	*p* Value	R	Lower	Upper	*p* Value
Energy (kcal)	0.57	0.37	0.72	<0.01	0.53	0.31	0.69	<0.001
Protein (g)	0.62	0.43	0.76	<0.001	0.53	0.32	0.69	<0.001
Fat (g)	0.43	0.19	0.62	<0.001	0.49	0.27	0.66	<0.001
Carbohydrate (g)	0.59	0.39	0.73	<0.001	0.48	0.25	0.65	<0.001
Dietary fiber (g)	0.45	0.22	0.63	<0.001	0.5	0.28	0.67	<0.001
Calcium (mg)	0.57	0.37	0.72	<0.001	0.53	0.31	0.69	<0.001
Iron (mg)	0.41	0.17	0.6	0.001	0.51	0.29	0.68	<0.001
Salt (g)	0.15	−0.12	0.39	0.27	0.37	0.13	0.58	<0.01
Vitamin B_1_ (mg)	0.44	0.22	0.63	<0.001	0.52	0.31	0.68	<0.001
Folate (μg)	0.53	0.32	0.69	<0.0001	0.53	0.32	0.7	<0.0001
Vitamin B_12_ (μg)	0.46	0.23	0.64	<0.001	0.4	0.16	0.59	<0.01
Vitamin D (μg)	0.37	0.13	0.57	<0.01	0.51	0.3	0.68	<0.01
Cholesterol (mg)	0.4	0.16	0.59	<0.01	0.39	0.14	0.59	<0.01
SFAs (g)	0.49	0.27	0.66	<0.0001	0.41	0.17	0.6	<0.01
MUFAs (g)	0.38	0.13	0.58	<0.01	0.47	0.24	0.64	<0.001
PUFAs (g)	0.34	0.1	0.55	<0.01	0.47	0.25	0.65	<0.001

Abbreviations: CI, 95% Confidence Interval; SFAs, saturated fatty acids; MUFAs, monounsaturated fatty acids; PUFAs, polyunsaturated fatty acids.

## Data Availability

The original contributions presented in the study are included in the article, further inquiries can be directed to the corresponding author.
